# Expired Patents

**Published:** 1907-01

**Authors:** 


### EXPIRED PATENTS.

November 26, 1906.

415788. Dentist's mouth mirror. G. F. Pease, Springfield, Mass.
415829. Tooth regulator. E. H. Angle, Minneapolis, Minn.
415983. Dentist's burr drill. E. T. Starr, Philadelphia, Pa.

December 3, 1906.

416426. Dental tool. S. Sibley, Philadelphia, Pa.

December 10, 1906.

416989. Dental instrument. L. C. Bryan, Basle, Switzerland.

The above list of patents are furnished by Davis & Davis, solicitors of American and foreign patents, Washington, D. C., and St. Paul Building, New York City.

